# Nutritional Value, Fatty Acid and Phytochemical Composition, and Antioxidant Properties of Mysore Fig (*Ficus drupacea* Thunb.) Fruits

**DOI:** 10.3390/foods13172845

**Published:** 2024-09-07

**Authors:** Hosakatte Niranjana Murthy, Guggalada Govardhana Yadav, Kadanthottu Sebastian Joseph, Sabha Khan H. S., Snehalata M. Magi, Yaser Hassan Dewir, Nóra Mendler-Drienyovszki

**Affiliations:** 1 Department of Botany, Karnatak University, Dharwad 580003, India; govardhanayadavgs@gmail.com (G.G.Y.); sabakhanhs231999@gmail.com (S.K.H.S.); snehalatam98@gmail.com (S.M.M.); 2 Department of Horticultural Science, Chungbuk National University, Cheongju 28644, Republic of Korea; 3 Department of Biotechnology, School of Advanced Sciences, KLE Technological University, Hubballi 580031, India; 4 Department of Life Sciences, Christ University, Bengaluru 560029, India; ksjoseph15@gmail.com; 5 Plant Production Department, College of Food and Agriculture Sciences, King Saud University, Riyadh 11451, Saudi Arabia; 6 Research Institute of Nyíregyháza, Institutes for Agricultural Research and Educational Farm (IAREF), University of Debrecen, P.O. Box 12, 4400 Nyíregyháza, Hungary; mendlerne@agr.unideb.hu

**Keywords:** antioxidant activity, *Ficus drupacea*, minerals, mysore fig, nutrients, proximate analysis

## Abstract

*Ficus drupacea* is a fruit-bearing tree that is distributed in Southeast Asia and Australia. The objective of this research was to ascertain the following with regard to ripened fruits: (i) their nutritional value, (ii) their mineral status, (iii) the fatty acid composition of fruit and seed oil, (iv) their phytochemical makeup, and (v) their antioxidant properties. The ripened fruits contained 3.21%, 3.25%, 0.92%, 1.47%, and 2.20% carbohydrate, protein, fat, ash, and fiber, respectively. Fruits had an energy content of 30.18 kcal/100 g. In terms of mineral content, the fruit was rich in potassium, magnesium, calcium, and nitrogen, with values of 21.03, 13.24, 11.07, and 4.13 mg/g DW. Iron, zinc, manganese, and boron had values of 686.67, 124.33, 114.40, and 35.78 µg/g DW, respectively. The contents of oxalate and phytate were 14.44 and 2.8 mg/g FW, respectively. The fruit and seed oil content were 0.67 and 8.07%, respectively, and the oil’s physicochemical properties were comparable to those of fig fruit and seed oils. Omega-3 (α-linolenic acid), omega-6 (linoleic acid), and omega-9 (oleic acid) fatty acids were abundant in the oils. Fruit extracts in acetone, methanol, and water have greater concentrations of phenolics, flavonoids, and alkaloids. The 2,2-diphenyl-2-picrylhydrazyl, total antioxidant activity, and ferric reducing antioxidant power assays demonstrated increased antioxidant activities in close correlation with the higher concentrations of phenolics, flavonoids, and alkaloids. The results of this study demonstrate that the fruits of *F. drupacea* are a strong source of nutrients and phytochemicals, and they merit more investigation and thought for possible uses.

## 1. Introduction

**Figure 1:**
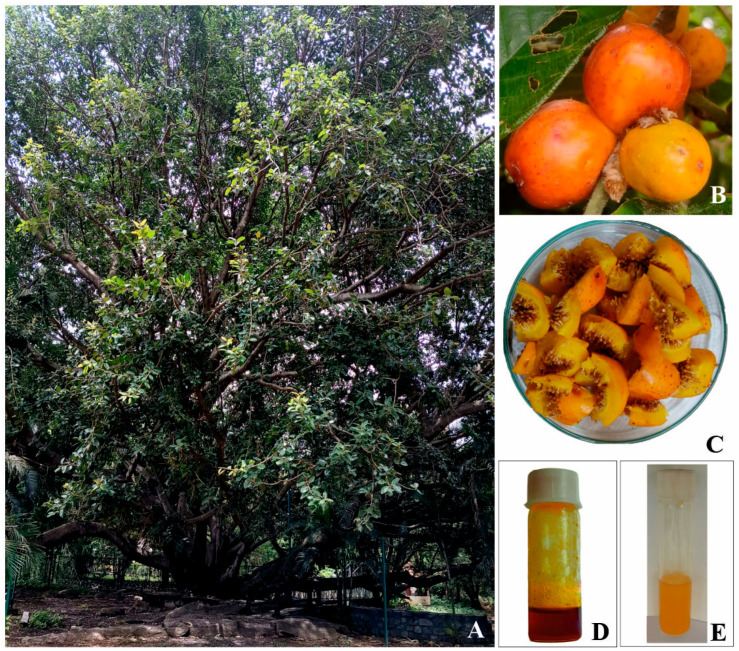
Morphology of *Ficus drupacea*. (**A**) Habit; (**B**) Fruits; (**C**) Fruits with seeds; (**D**) Fruit oil; (**E**) Seed oil.

*Ficus drupacea* Thunb. (syn. *Ficus drupacea* Thunb. var. *pubescens* (Roth) Corner, Family: Moraceae), commonly known as ‘Mysore fig’ or ‘brown-woolly fig’, is a fruit-bearing tropical tree native to Southeast Asia and Northeast Australia [1]. It possesses a dense woolly pubescence; therefore, it is designated as a ‘brown-woolly fig’, and it grows to a height of 10–30 m (Figure 1A). Trees possess aerial roots arising in tufts from the stout branches. The bark is greyish brown and exudates milky latex. The syconium is the type of inflorescence that is formed by an enlarged, fleshy, hollow receptacle with numerous female and male flowers on the inside surface, and it subsequently develops into multiple and accessory fruits. Fruits are ovoid to cylindrical, yellow to red, and edible (Figure 1B) [2]. *Ficus drupacea* is known for its medicinal properties and is used for the treatment of various ailments, including malaria and sinusitis [3]. *Ficus drupacea* stem bark has been shown in recent studies to possess antifungal, antibacterial, and anticancer properties. Yessoufou et al. [3] isolated and identified several phytochemicals from the bark, including β-amyrin, β-sitosterol-3-O-β-D-glucopyranoside, 5-O-methyllatifolin, oleanolic acid, epifriedelanol, friedelin, and epilupeol acetate. Manjuprasanna et al. [4] have reported the isolation of a cysteine protease called Drupin from latex, which has exhibited hemostatic characteristics.

Common fig (*Ficus carica* L.) fruits are highly commercially valuable and provide essential nutrients for human nutrition, including antioxidants, minerals, vitamins, carbohydrates, and amino acids [5]. Additionally, *Ficus carica* has several phytochemicals that aid in the treatment of many diseases, such as obesity, diabetes, cancer, neurodegenerative diseases, and cardiovascular diseases [6,7]. *Ficus drupacea* is collected in the wild and used as a food source, medicinally, and as a fiber source [2]. Even though there is a lot of potential for human use, this species remains underappreciated and ignored. The nutritional, mineral, and phytochemical properties are not known. Consequently, in the current investigation, we aimed to examine the nutritional content of *Ficus drupacea* fruits. We also evaluated the antioxidant properties of fruit extract, conducted mineral and phytochemical analysis, and examined the fatty acid composition of fruit and seed oil.

## 2. Materials and Methods

### 2.1. Plant Materials and Sample Preparation

*Ficus drupacea* fruits were collected at random from five trees in the Haveri district of Karnataka, India, which is located near Shiggavi (14.992726 N, 75.183157 E). The fruits were divided into two groups; from one, only the seeds were removed for study, while from the other, the entire fruit was used. To eliminate the moisture content, the fruits were cut into pieces (Figure 1C) and dried in an oven at 40 ± 2 °C. Following the drying process, the fruits and seeds were ground into a powder using a mechanical grinder and kept at room temperature in airtight polythene bags until additional examination.

### 2.2. Chemicals

Chemicals, such as Folin–Cicalteau reagent, BF3-methanol, and anthrone, and standard chemicals, such as bovine serum albumin, glucose, and sodium phytate, used in this study were procured from Himedia laboratories, Mumbai, India, whereas heptadecanoic acid was purchased from Sigma-Aldrich, Bengaluru, India. All the other chemicals and solvents used were of analytical grade.

### 2.3. Proximate Analysis

The fruit’s moisture, lipid, ash, and protein levels were examined using AOAC [8] procedures. To put it briefly, the weight difference of the oven-dried sample at 102 °C was recorded for six hours to measure the moisture content gravimetrically. Gravimetric analysis was used to determine the oil content of the sample’s fruit and seeds (Figure 1D,E). Using a Soxhlet apparatus at 65 ± 2 °C for 8 h, the finely crushed powder of fruit and seeds was extracted with petroleum ether (40–60 °C) to obtain the oil. The solvent fraction was then evaporated using a rotary evaporator (Buchi, Rotavapor R-100, Flawil, Switzerland). To eliminate any remnants of the solvents, the oil was maintained at 40 ± 2 °C in an oven until its weight stabilized. Additionally, the ash content of the samples was ascertained by igniting the oven-dried samples in the muffle furnace at 750 °C. The oil content was ascertained gravimetrically and kept at −20 °C until additional analysis. Then, 500 mg of defatted samples were ground with 5–10 mL of buffer for the protein content assay. The known volume of extracted material was mixed with 4.5 mL of a 2% sodium carbonate solution. The sodium carbonate solution was made with 0.1 N sodium hydroxide (50 mL), 0.5% copper sulphate (1 mL), and 1% potassium sodium tartrate. Following a 10 min incubation period, tubes were supplemented with 0.5 mL of Folin–Cicalteau reagent and maintained at room temperature in the dark for 30 min to produce a blue color. The spectrophotometric method was used to measure the color developed at 660 nm. The standard utilized was bovine serum albumin. The anthrone reagent method was used to quantify the amount of carbohydrates, and acid and alkali digestion was used to determine the fiber content of the samples. Atwater-specific conversion factors were used to compute the energy value, as reported by the FAO [9].

### 2.4. Analysis of Mineral Composition

An air or acetylene flame was used with a NOVA 400 atomic absorption spectrophotometer (type Analytic Jena, Jena, Germany) to analyze potassium, phosphorus, sulfur, sodium, calcium, boron, manganese, magnesium, copper, iron, and zinc. Using hollow cathode lamps, the absorbance was measured [8]. To measure nitrogen, a two-step digestion–UV spectrophotometric method was employed [10].

### 2.5. Physicochemical Estimation of Seed Oil

After four hours of incubation at room temperature, the extracted oil’s color and physical condition were assessed. An Abbe refractometer and a specific gravity bottle were used to calculate the oil’s density and refractive index, respectively. The AOCS [11] methods were followed to evaluate the free fatty acid (FFA) concentration, peroxide value (PV), iodine value, and unsaponification values. Spectrophotometric methods were employed to determine the content of carotenoids and lignans, as stated by Manasa et al. [12].

### 2.6. Fatty Acid Profiling

The esterification procedure was used to produce fatty acid methyl esters (FAMEs) following AOCS [11] recommendations. In this process, 1 mL of BF3-methanol was mixed with 15 mg of the oil sample, and the mixture was incubated for 30 min at 60 °C. Without delay, the reaction tubes were placed in an ice bath and kept there for five minutes. The mixture was then vortexed after 1 mL of hexane and distilled water were added. After that, the undisturbed methyl esters that made up the top layer were transferred to GC vials. The internal standard was heptadecanoic acid. The identification of FAMEs was accomplished, as reported by Manasa et al. [12], using a GC-MS (PerkinElmer, Turbo-mass Gold, mass spectrometer, Waltham, MA, USA) equipped with a flame ionization detector (FID) and a fused silica Rtx-2330 column (Restek manufactured, 30 m, 90.32 mm ID, and 0.20 mm film thickness, Bellefonte, PA, USA). Nitrogen was used as the carrier gas, the injector port was kept at 230 °C, and the detector temperature was set at 250 °C. The column temperature was 120 °C at first, then it rose gradually over the course of 20 min to 220 °C, and it remained there for an additional 10 min. The fragmentation pattern and retention time were compared to industry norms and the NIST library to identify FAMEs.

### 2.7. Analysis of Anti-Nutritional Factors

#### 2.7.1. Estimation of Phytate

Next, 10 mL of 2.4% HCl was used to extract the defatted seed cake (0.5 g) for 16 h with continuous agitation. The mixture was then filtered. After adding 1 g of NaCl to the filtrate, it was vigorously agitated for 20 min. After centrifuging the mixture for 20 min at 10 °C at 1000× *g*, the known volume of the supernatant was diluted to 3 mL with distilled water, and Wade’s reagent (0.03% FeCl_3_·6H_2_O + 0.3% sulfosalicylic acid) was added. With a UV-Vis spectrophotometer, the color-developed absorbance was measured at 500 nm. A control was created without any sample additions. The standard utilized was sodium phytate [13]. The linear equation for the analysis was y = 0.0012x + 0.0165.

#### 2.7.2. Estimation of Oxalate

Oxalate was determined using the method of Salgado et al. [14]. After adding 2 g of defatted seed cake to 190 mL of distilled water and 10 mL of 6 N HCl, the mixture was heated for 4 h at 90 °C in a water bath. After filtering the mixture and adjusting its volume to 250 mL, 50 mL of this solution was titrated against concentrated ammonia in the presence of a methyl orange indicator. It was then heated to 95 °C, and 10 mL of 5% CaCl_2_ was added. For the calcium oxalate precipitation, 6 N NH_4_OH was added after 10 min, and the color change was monitored and maintained overnight. After filtering and dissolving the precipitate in hot sulfuric acid, 125 mL of the filtrate was prepared, heated to 95 °C, and titrated against 0.05 N KMnO_4_. To calculate oxalate, the following formula was used:

Oxalate (%) = (mL KMnO_4_) (0.05) (45.02) × (100) (5)/(1000) (wt. of the sample in grams)

### 2.8. Quantitative Phytochemical Analysis

#### 2.8.1. Extraction Procedure

Three separate solvents were used to extract the defatted fruit powder during an eight-hour period using a Soxhlet apparatus: acetone, methanol, and water, in increasing order of their polarity (acetone < methanol < water). The next solvent was made from the residue left over from the previous solvent extraction. Following the extraction process, the solvents were removed using a rotary evaporator (Buchi, Rotavapor R-100, Flawil, Switzerland). The extracts were then stored at 4 °C until they were needed, and any leftover solvents were removed by heating them in an oven at 40 ± 2 °C.

#### 2.8.2. Quantification of Phenolics

A slightly modified version of the Murthy et al. [15] method was used to calculate the total phenolics. In summary, 0.5 mL of the extract (0.33 mg/mL concentration) was diluted to 3 mL using distilled water, and 0.1 mL of 2 N Folin–Ciocalteau reagent was added. Following a 6 min incubation period, 0.5 mL of 20% sodium carbonate (Na_2_CO_3_) was added. A UV-Vis (ultraviolet-visible) spectrophotometer (Hitachi U-3310, Ibaraki, Japan) was used to measure the developed color absorbance at 760 nm after the tubes had been left in a warm water bath for 30 min. The standard was gallic acid. The linear equation for the analysis was y = 0.0208x + 0.0155.

#### 2.8.3. Quantification of Flavonoids

The flavonoid concentration in each of the extracts was estimated using the method suggested by Dalawai et al. [16]. In summary, 0.5 mL of extract (1 mg/mL concentration) was diluted to 3 mL using distilled water, then 0.15 mL of NaNO_3_ was added and the mixture was incubated for 5 min at room temperature. After adding 0.3 mL of 10% AlCl_3_ and 2 mL of 1 M NaOH, the solutions were vortexed, and the absorbance at 510 nm was measured. The standard was quercetin. The linear equation for the analysis was y = 0.0066x + 0.0003.

#### 2.8.4. Quantification of Alkaloids

The Murthy et al. [15] approach was used to quantitatively measure the alkaloid content: 6.98 mg of bromocresol green powder was dissolved in 0.3 mL of NaOH to prepare the bromocresol green solution, which was then finally diluted with distilled water to a final volume of 100 mL. Then, 5 mL of the previously prepared bromocresol green solution was added to 1.0 mL (5 mg/mL concentration) of a known quantity of material. Next, 5 mL of phosphate buffer (2 M sodium phosphate and 0.2 M citric acid with pH adjusted to 4.7) was added. After adding and vigorously shaking 5 mL of chloroform, the absorbance at 470 nm was measured, and the chloroform layer was recovered. The standard practice was to use atropine. The linear equation for the analysis was y = 0.0039x + 0.0032.

### 2.9. Antioxidant Activity

#### 2.9.1. Determination of Antioxidant Activity Using 2,2′-Diphenyl-1-Picrylhydrazyl (DPPH) Radical Scavenging Method

The 0.1 mL extracts of various extract concentrations (3 mg/mL for acetone and methanol extracts and 0.5 mg/mL for water extract) were mixed with 1.9 mL of a 0.1 mM DPPH solution made in methanol. After shaking the tubes well, they were dark-incubated for fifteen minutes. Using a spectrophotometer set at 517 nm, the color intensity of the DPPH solution was determined. Gallic acid was used as the standard, and activity was expressed in milligrams of gallic acid equivalent (GAE)/gram of extract, according to Yadav et al. [17]. The linear equation for the analysis was y = 0.0618x − 0.0023.

#### 2.9.2. Total Antioxidant Activity (TAA)

The phosphomolebdenum method was used to carry out the total antioxidant assay [17]. The 0.15 mL extracts at different concentrations (3 mg/mL for acetone and methanol extracts and 0.1 mg/mL for water extract) were combined with 1.5 mL of the reagent solution, which comprised 0.6 M sulfuric acid, 28 mM sodium phosphate, and 4 mM ammonium molybdate. The hue developed in the tubes was measured at 695 nm following 90 min of incubation at 95 °C. Ascorbic acid was used as the reference, and the activity was expressed in milligrams of ascorbic acid equivalent per gram (mg AAE/g) of extract. The linear equation for the analysis was y = 0.0078x − 0.0007.

#### 2.9.3. Ferric Reducing Antioxidant Power (FRAP)

Using the Murthy et al. [18] methodology, the FRAP assay was conducted. First, 0.1 mL of the extract (3 mg/mL for acetone and methanol extracts and 0.1 mg/mL for water extract) was mixed with 3 mL of FRAP reagent, which contained 300 mM acetate buffer (pH 3.6), 10 mM of TPTZ (2,4,6-tripyridyl-s-triazine) in 40 mM HCl, and 20 mM FeCl_3_·6H_2_O (10:1:1). The absorbance at 593 nm was measured in relation to a blank solution after the sample and FRAP reagent-filled tubes were vortexed and allowed to stand for six minutes at room temperature. The activity of the extracts is represented as mg ascorbic acid equivalent (AAE)/g extract, with ascorbic acid serving as the standard. The linear equation for the analysis was y = 0.0526x + 0.0373.

### 2.10. Analysis of Data and Statistical Treatment

Each experiment was conducted three times, and the results were reported as mean values with standard errors. Descriptive statistics, including the mean and standard error, were calculated using Microsoft Excel 2019. The statistical significance of the differences between mean values was assessed using Duncan’s multiple range test at *p* < 0.05. All the statistical analyses were performed using SAS 9.4 software (SAS Institute Inc., Cary, NC, USA).

## 3. Results and Discussion

### 3.1. Nutritional Value

One of the most prevalent fruits in the Mediterranean region, the common fig (*F. carica*), is a significant crop grown all over the world [19]. According to Alzaharni et al. [5], the approximate composition of a common fresh fig is as follows: 79.9–88.1% moisture, 7.60–20.0% carbohydrate, 0.53–1.30% fat, 0.60–4.00% ash, 2.10–2.20% fiber, and an overall energy value of 37.0 kcal/100 g. According to the current investigation, the *F. drupacea* fruits’ approximate composition was 87.99 ± 0.20% moisture, 3.21 ± 0.15% carbohydrate, 3.25 ± 0.26% protein, 0.92 ± 0.15 fat, 1.47 ± 0.04% ash, 2.20 ± 0.25% fiber, and 30.18 kcal/100 g of energy, which is similar to that of common figs (Table 1). The fruits of *F. auriculata* were found to have the following nutritional values: 15.22 ± 3.4% ash, 1.82 ± 1.0% fiber, 0.01 ± 0% fat, 3.19 ± 1.0% protein, 35.42 ± 4.5% carbohydrate, and 141.68 kcal/100 g energy, respectively [20]. On the other hand, the approximate contents of *F. hispida* fruits were 1.71 ± 1.0 fiber, 14.94 ± 3.1% ash, 3.11 ± 1.0% protein, 36.33 ± 5.6% moisture, 43.86 ± 5.3% carbohydrate, and 175.44 kcal/100 g, in that order. According to Rusmadi et al. [20], fruits of *F. fistulosa* have 44.0 ± 5.3 moisture, 33.66 ± 4.4% carbohydrate, 2.90 ± 1.0% protein, 0.02 ± 0% fat, 15.91 ± 3.6% ash, 1.61 ± 1.0% fiber, and 142.29 kcal/100 g energy. The *F. amplissima* fruits had nutritional values of 83.29%, 15.45%, 1.81%, 0.63%, 0.82%, and 0.81% for moisture, protein, fat, ash, and fiber, respectively, and 53.09 kcal/100 g for energy [21]. Variations in the environmental conditions of agriculture and species diversity may have contributed to the variation in the nutritional qualities of different fruits.

### 3.2. Mineral Composition

Fruits are an excellent source of minerals and are essential for maintaining many physiological processes in the human body, such as the growth of bones, muscles, and nerves, as well as the control of the body’s water balance [22]. *F. carica* is rich in minerals, which help the body function normally by supplying calcium, iron, phosphorus, potassium, and sodium [5]. Among the several minerals contained in *F. carica* that support healthy bone development are iron and strontium [23]. Fruits of many *Ficus* species are well known for having a mineral-rich makeup, and *F. drupacea* fruits are no exception (Table 2). Particularly, fruits of *F. drupacea* have high concentrations of potassium, magnesium, calcium, and nitrogen (21.03, 13.24, 11.07, and 4.13 mg/g DW, respectively). Furthermore, the concentrations of phosphorus, sulphur, and sodium were 1.64, 1.02, and 0.54 mg/g DW, respectively. Different microelements with notable concentrations include iron, zinc, manganese, boron, and copper (686.67, 124.33, 114.40, 35.78, and 13.98 µg/g DW, respectively) (Table 2).

*Ficus begalensis*, *F. recemosa*, *F. religosa*, *F. palmata*, *F. microcorpa*, *F. johannis*, *F. sarmentosa*, *F. hispida*, and *F. auriculata* were among the fruits of various *Ficus* species that Khan et al. [24] analyzed elementally. The range of potassium, magnesium, and calcium in these fruits was 57.3–11.29, 7.26–3.83, and 24.9–24.08 mg/g DW in various species. In contrast, the ranges for iron, manganese, copper, and zinc in different species were 2.9–0.51, 32.53–10.01, 6.96–1.00, and 20.49–5.05 mg/g DW. Such variation is evident in the variety of *Ficus* species, and it also depends on the mineral nutrition levels and soil conditions in the plant’s growing medium. Many elements, including potassium, calcium, manganese, iron, copper, and zinc, are essential for the synthesis of secondary metabolites, which give plants their medicinal and pharmacological properties and enable the plants to fight disease. Studies show that calcium helps prevent osteoporosis and the fractures that result from it. It takes magnesium to prevent cardiovascular diseases. Furthermore, phosphorus is required for the development and maintenance of bodily structures and cells [22]. The fruits of *F. drupacea* have the highest concentration of iron (686.67 µg/g DW). Iron is linked to angiotensin-converting enzyme inhibitors and is a necessary mineral for the prevention of anemia [25]. In contrast, several trace elements can both prevent and treat disease [26]. The aforementioned findings demonstrate that *F. drupacea* fruits are edible and a rich source of the necessary mineral components for human health.

### 3.3. Antinutrient Composition

Table 3 lists the antinutritional components of *F. drupacea* fruits, including their oxalate and phytate contents. The phytate concentration was 2.8 mg/g FW, which is lower than the quantity reported in *Rourea minor* [27] but equivalent to phytate values found in fruits of the *Balanitis aegyptica* species (0.06–1.82 mg/g) [28]. Phytate decreases the digestibility of amino acids and forms complexes with phosphorous, calcium, iron, and zinc to obstruct their absorption. As a result, the body can easily no longer access these nutrients. The phytate levels found in this investigation, however, were less than the 10–60 mg/g that have been linked to issues with mineral bioavailability [29]. The fruit of *F. drupacea* contained 14.44 mg/g FW of oxalate. It is well known that oxalate, particularly at concentrations of roughly 45 g/100 g, inhibits the renal absorption of calcium [30]. Nonetheless, the values found in this investigation are significantly lower than the values that are thought to be hazardous. This implies that consuming the fruits might not provide any issues with mineral absorption.

### 3.4. Characterization of Fruit and Seed Oil

For human health, edible oil is a vital dietary resource. Edible oil can be extracted from plants using their fruits or seeds. The acquired oils are either discovered to have medicinal and cooking uses or they are utilized for edible purposes. Due to rising demand for vegetable oils in the chemical industry, animal feed, pharmaceutical, and other fields, oil-yielding plants have garnered increased attention recently. Rapid population growth and resource depletion present problems for the entire planet. Investigating substitute sources of edible oil is crucial [31]. Different plant species or plant parts differ significantly in terms of their oil concentration, composition, and qualities [32]. To extract oils from fruits and seeds, we investigated the oil content of *F. drupacea* in the current study. Fruits of *F. drupacea* have less oil (0.67%) (Figure 1D), but seeds have a substantial amount of oil (8.07%) (Figure 1E). Table 4 lists the fruit and seed oil’s physicochemical characteristics. In contrast to the seed oil, which was crimson yellow and liquid, the fruit oil had a blood-red hue and was viscous at room temperature. The refractive index of *F. drupacea* fruit oil is 1.510, while that of the seed oil is 1.498. These values are similar to those of common edible oils, such as corn oil (1.473) and soybean oil (1.472), and are correlated with the molecular weight, degree of unsaturation, and length of the fatty acid chain of the oil [33]. *F. drupacea* fruit and seed oils have densities of 0.921 and 0.931 g/cm^3^, respectively, which are similar to corn oil’s value of 0.917 g/cm^3^ [33]. Essential characteristics of edible seed oils are their peroxide and free fatty acid levels. Edible natural oils with less than 5% free fatty acid concentration are a possibility [34,35]. *F. drupacea* fruit and seed oil had a lower free fatty acid concentration (1.03 and 1.55%, respectively) than mustard oil, but it was nevertheless comparable to several commercial oils like sesame oil [36]. The fruit and seed oils of *F. drupacea* have peroxide values of 19.79 and 19.92 meq O_2_/kg (Table 4), which are lower than those of various commercial edible oils, including sunflower, peanut, olive, mustard, and rape seed oils [37] and *Balanites roxhbergii* crude oil [17]. As a measure of an oil’s unsaturation level, the iodine value for *F. drupacea* fruit and seed oil was 88.21 and 154.03 I_2_/100 g, respectively. These values are higher than those of edible refined oils like olive oil (80.03 I_2_/100 g) and mustard oil (94.95 I_2_/100 g) [37]. Table 4 shows that the fruit and seed oils of *F. drupacea* had unsaponification values of 15.28% and 2.80%, respectively, indicating the presence of nutraceuticals in the oils besides fatty acids. The results are greater than those of mustard oil (1.01%) and olive oil (1.23%) [37]. The formation of cataracts and age-related macular degeneration are both slowed down by the vital natural pigments called carotenoids. Additionally, they have antioxidant properties that enhance cognitive and cardiovascular health [38]. The fruit and seed oils from *F. drupacea* had a greater carotenoid concentration (1048.73 and 36.64 mg/kg, respectively), higher than apple, apricot, and blackberry fruit oils (15.80, 66.80, and 13.30 mg/kg, respectively) [39]. Lignans are polyphenols with biological activities that include preventive properties against diseases including hormone-dependent cancers and cardiovascular disorders. They also exhibit prooxidant and antioxidant actions [40]. *F. drupacea* fruit and seed oils had lignan contents of 0.59 and 0.83%, respectively, which are lower than *Erythrina stricta* seed oil (165.47 mg/100 g) [35].

**Figure 2:**
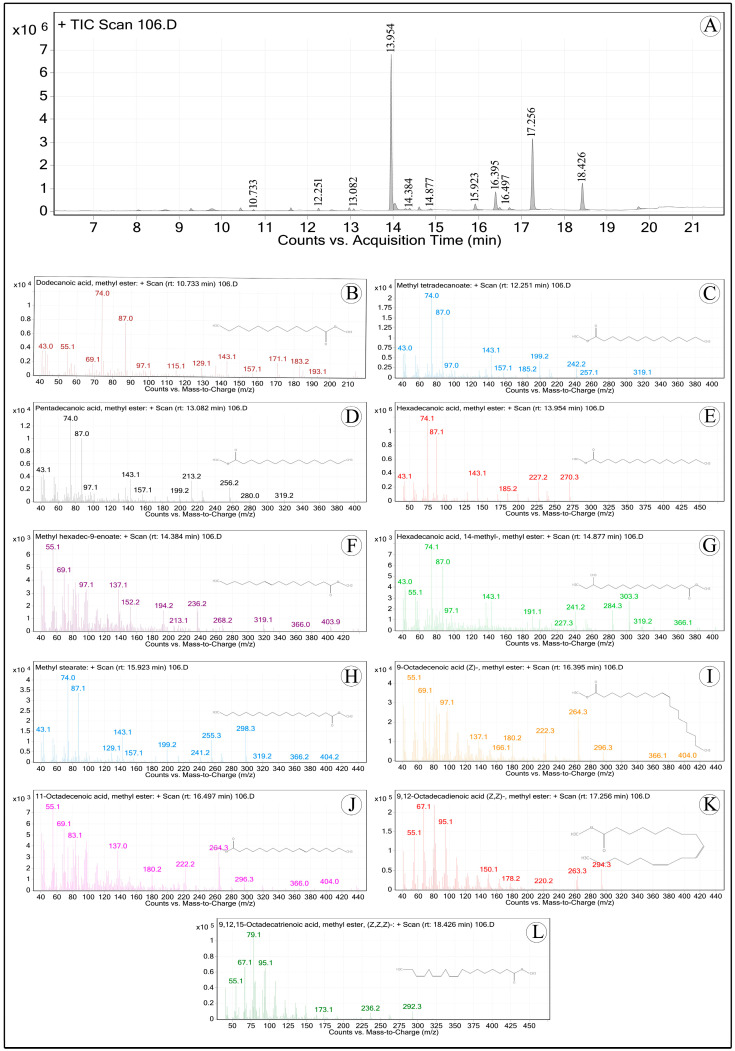
(**A**) GC-MS chromatograms of *Ficus drupacea* pulp oil fatty acid methyl esters (FAMEs); B-L. Mass spectra of major FAMEs of analyzed fatty acids. (**B**) Methyl laurate; (**C**) Methyl myristate; (**D**) Pentadecanoic acid, methyl ester; (**E**) Methyl palmitate; (**F**) Methyl hexadic-9-enoate; (**G**) Hexadecanoic acid, 14-methyl-, methyl ester; (**H**) Methyl stearate; (**I**) Methyl oleate; (**J**) Methyl vaccinate; (**K**) Methyl linoleate; (**L**) Methyl linolenate.

Table 5 shows the fatty acid content of *F. drupacea*’s fruit and seed oils, and Figure 2 displays the GC-MS chromatograms analyzed for fatty acid profiling. Fruit oil contained the following main fatty acids: oleic acid (7.07%), linoleic acid (28.61%), α-linolenic acid (11.91%), and palmitic acid (46.08%), and 50.80:8.68:40.52% was the overall concentration of monounsaturated, polyunsaturated, and saturated fatty acids (Table 5). These results contrast from the fatty acid composition of fruit oil from *F. carica*, which contains oleic acid (10.0%), palmitic acid (14.0%), linoleic acid (21.0%), and α-linolenic acid (53%) [5]. On the other hand, *F. exasperata* fruit oil had a relatively lower amount of saturated fatty acids, such as stearic acid (9.10%) and palmitic acid (7.32%), and a higher amount of unsaturated fatty acids, such as linoleic acid (54.54%) and oleic acid (18.89%) [41].

**Figure 3:**
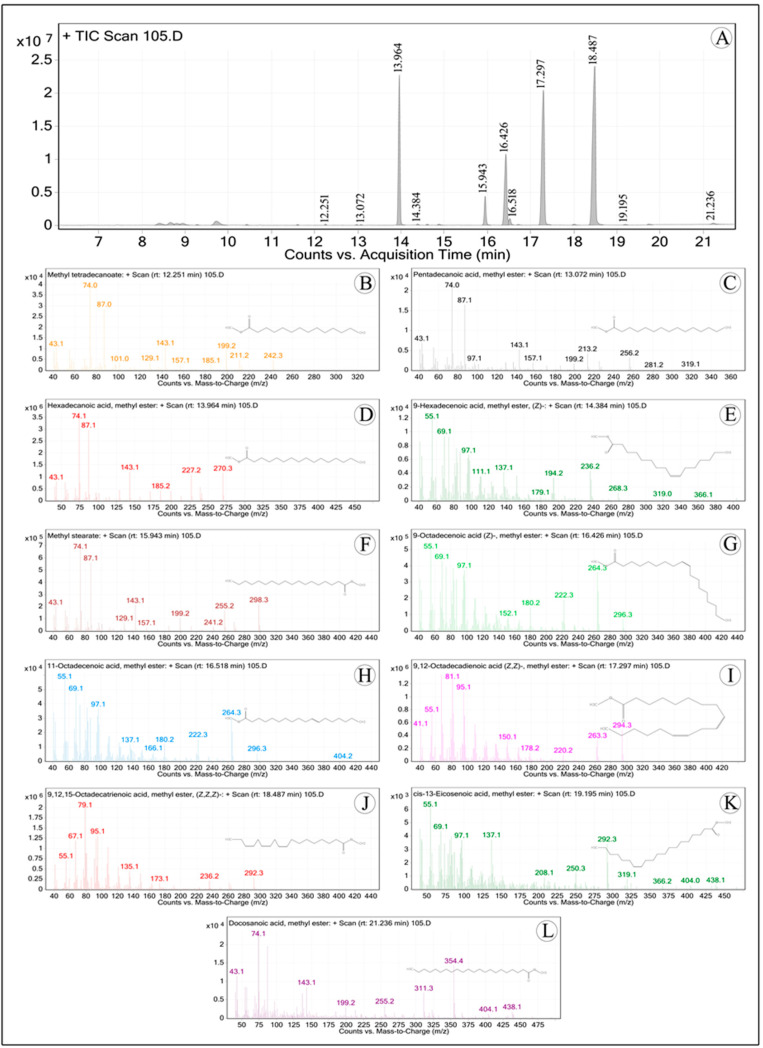
(**A**) GC-MS chromatograms of *Ficus drupacea* seed oil fatty acid methyl esters (FAMEs); B-L. Mass spectra of major FAMEs of analyzed fatty acids. (**B**) Methyl myristate; (**C**) Pentadecanoic acid, methyl ester; (**D**) Methyl palmitate; (**E**) Methyl hexadic-9-enoate; (**F**) Methyl stearate; (**G**) Methyl oleate; (**H**) Methyl vaccinate; (**I**) Methyl linoleate; (**J**) Methyl linolenate; (**K**) Methyl paullinate; (**L**) Methyl behenate.

The major fatty acids found in the seed oil *F. drupacea* in the present study were oleic acid (12.63%), palmitic acid (18.16%), α-linolenic acid (36.87%), and linoleic acid (26.81%) (Table 5; Figure 3). The proportions of saturated fat, monounsaturated fat, and polyunsaturated fat were 22.46%, 13.87%, and 66.68%, respectively. These findings were similar to those of seed oil *F. carica*, where the primary fatty acids α-linolenic acid (41.80–37.87%), linoleic acid (37.95–31.80%), and palmitic acid (7.40–3.58%) were found in several accessions [42]. Consistent with the aforementioned findings, Baygeldi et al. [43] detected noteworthy concentrations of omega-3 (α-linolenic acid, 41.75%), omega-6 (linoleic acid, 30.9%), and omega-9 (oleic acid,15.98%). The essential fatty acids α-linolenic and linoleic can be found in the seed oils of *F. carica* and *F. drupacea*, which can be employed in nutraceutical products.

### 3.5. Phytochemical Composition

Three important phytochemical groups—phenolics, flavonoids, and alkaloids—were examined in the fruits of *F. drupacea* through extraction using various solvents. The findings are shown in Table 6. Methanol yielded the highest extract (259.98 mg/100 g) of all the extracts, compared to acetone (20.86 mg/100 g) and water extract (3.96 mg/100 g). Fruits of *F. drupacea* are better suited for the methanol extraction method, which has the highest yield. Several researchers employed a similar approach and discovered that it was helpful to extract phytochemicals from plant material using a variety of solvents [18].

The antioxidant activity of phenolics is responsible for their anti-inflammatory, anti-tumor, anticancer, antidiabetic, antibacterial, and anti-osteoporosis characteristics [44]. The total phenolic content of six underutilized fruits (*Carrisa carandas*, *Dovyalis hebecarpa*, *Flocourtia indica*, *Malphighia emerginata*, *Slacia chinensis*, and *Syzygium indica*) was examined by Perera et al. [45] and the results ranged from 6.77 to 10.29 mg GAE/g on a fresh weight basis. The total phenolic content of *F. drupacea* fruit extracts was 60.12, 25.78, and 70.50 mg GAE/g extract in acetone, methanol, and water extract, respectively, indicating the fruits’ high power for preventing free radicals that cause health problems in the body. According to Ullah et al. [46], flavonoids are an additional class of antioxidants that help regulate inflammatory, cardiovascular, and carcinogenic issues in humans. The *Rourea minor* fruit extracts were tested with acetone, methanol, water, and 70% methanol extract. The total flavonoid content was 1.37, 1.27, 3.25, and 1.61 mg QE/g DW, respectively [27]. Total flavonoid concentrations in the fruits of *F. drupacea* were 16.53, 5.88, and 7.12 mg QE/g extract, respectively. These values were greater than those of the following wild fruits: *Baccaurea sapida*, *Docynia indica*, *Elaeagnus latifolia*, *Elaeagnus pyriformis*, *Haematocarpus validus*, *Myrica esculanta*, *Myrica nagi*, *Prunus nepalensis*, *Pyrus pashia.* In these fruits, the flavonoid content ranged from 2.77 mg QE/g DW in *Pyrus pashia* to 5.46 mg QE/g DW in *Haematocarpus validus* [47]. Another significant class of secondary metabolites found in plants is called the alkaloids, and they are widely used as antibacterial, anticancer, cardioprotective, antidiabetic, and neuroprotective medicines [48]. The total alkaloid concentrations of longan fruits were estimated by Tang et al. [49], who reported 1.67, 6.44, and 7.40 mg HE/g for the pulp, pericarp, and seed, respectively. The acetone, methanol, and water extract of *F. drupacea* fruits yielded alkaloid contents of 3.76, 1.37, and 0.04 mg AE/g extract, respectively. These values were significantly lower than those of longan fruits. These findings show that the content of alkaloids present in different fruits varies by species.

### 3.6. Antioxidant Activities

Antioxidants are essential for preserving human health because they protect the body from oxidative stress and prevent a number of illnesses, including cancer, autoimmune disorders, Parkinson’s disease, and Alzheimer’s. Bioactive chemicals obtained from plants protect cells against oxidative damage by their inhibition or interaction with reactive oxygen species and free radicals [44,46]. The fruit extract of *F. drupacea* exhibited a noteworthy phytochemical presence, as evidenced by the antioxidant activities evaluated using three conventional in vitro methods: FRAP assay, total antioxidant activity, and DPPH radical scavenging activity. The findings are displayed in Table 7.

Acetone extract had the highest activity among the studied extracts, including methanol and water, as determined by TAA and FRAP methods. The DPPH radical scavenging activity of the water extract was 4.82 mg GAE/g, but the acetone and methanol extracts showed 3.81 and 2.54 mg GAE/g extract, respectively. TAA was measured as follows: 139.5 and 97.99 mg AAE/g extract in the case of water and methanol extracts, and 3103 mg AAE/g extract in the case of acetone extract. The acetone extract had a FRAP activity of 965.21 mg AAE, while the methanol and water extracts had FRAP activities of 10.66 and 38.14 mg AAE/g, respectively. Similar to the current findings, Arunachalam et al. [21] found that *Ficus amplissima* extracts prepared in hot water, petroleum ether, chloroform, acetone, and methanol have extremely strong antioxidant activity when tested utilizing TAA and FRAP assay techniques. On the other hand, Rajesh et al. [50] have suggested that the high phenolic content in the *Ficus racemosa* samples may be the cause of their ability to scavenge free radicals. Numerous other studies using a range of antioxidant assays have effectively established the antioxidant qualities of wild and underutilized fruits. For example, the unripe pulp outperformed the matured one in *Diospyros chloroxylon* when it came to scavenging DPPH radicals, while acetone extracts outperformed methanol and water extracts [15]. As demonstrated by the DPPH and FRAP tests, the antioxidant activity of black chokeberry, raspberry, blackberry, and mulberry was attributed to their phenolic components [51].

## 4. Conclusions

This study showed that *Ficus drupacea* fruits are nutrient-dense, containing ash, fiber, protein, fat, and important minerals. The fruits have comparatively low levels of antinutrients. α-linolenic acid, oleic acid, and linoleic acid were the main fatty acids in the fruit and seed oil, which have properties similar to those of figs. The fruits also exhibit strong free radical scavenging properties due to their abundance of phytochemicals, which include flavonoids, phenolics, alkaloids in acetone, methanol, and water extracts of fruits. The aforementioned findings demonstrate that *Ficus drupacea* fruits are valuable for food uses. The current study’s merits include providing information on the nutritional value, oil content, fatty acid composition, phytochemical status, and antioxidant status of the underutilized fruit, *F. drupacea*. However, further investigation would be required to examine the additional beneficial phytochemicals, particularly pigments, and to precisely quantify the specific phenolic, flavonoid, and associated antioxidant activities of these compounds.

## Figures and Tables

**Table 1:** Proximate composition of *Ficus drupacea* fruit.

Component	Composition (%)
Moisture	87.99 ± 0.20 ^z^
Carbohydrate	3.21 ± 0.15
Protein	3.25 ± 0.26
Fat	0.92 ± 0.15
Ash	1.47 ± 0.04
Fiber	2.20 ± 0.25
Energy (kcal/100 g)	30.18

^z^ Each value represents the mean ± standard error of three replicates.

**Table 2:** Mineral composition of *Ficus drupacea* fruit.

Mineral	Composition
Microelements (mg/g DW)	
Nitrogen	4.13 ± 0.06 ^z^
Phosphorous	1.64 ± 0.02
Potassium	21.03 ± 0.12
Sulphur	1.02 ± 0.03
Sodium	0.54 ± 0.01
Calcium	11.07 ± 0.12
Magnesium	13.24 ± 0.09
Microelements (µg/g DW)	
Boron	35.78 ± 0.44
Zinc	124.33 ± 0.88
Iron	686.67 ± 8.82
Manganese	114.40 ± 0.29
Copper	13.93 ± 0.20

^z^ Each value represents the mean ± standard error of three replicates.

**Table 3:** Anti-nutritional factors of *Ficus drupacea* fruit.

Factor	Composition (mg/g FW)
Phytate	2.80 ± 0.01 ^z^
Oxalate	14.44 ± 0.06

^z^ Each value represents the mean ± standard error of three replicates.

**Table 4:** Physicochemical characteristics of *Ficus drupacea* fruit and seed oil.

Parameter	Fruit Oil ^z^	Seed Oil ^z^
Oil yield (%)	0.67 ± 0.15 ^b^	8.07 ^a^
Color	Blood red	Crimson yellow
State at room temperature	Solid	Liquid
Refractive index	1.510 ± 0.01 ^a^	1.498 ± 0.01 ^a^
Density (g/cm^3^)	0.921 ± 0.01 ^a^	0.931 ± 0.01 ^a^
Free fatty acid content (%)	1.03 ± 0.02 ^a^	1.55 ± 0.08 ^a^
Peroxide value (meq O_2_/kg)	19.79 ± 1.46 ^a^	19.92 ± 0.77 ^a^
Iodine value (I_2_/100 g)	88.21 ± 0.12 ^b^	154.03 ± 2.30 ^a^
Unsaponification value (%)	15.28 ± 0.10 ^a^	2.80 ± 0.06 ^b^
Carotenoids (mg/kg)	1048.73 ± 39.87 ^a^	36.64 ± 3.32 ^b^
Lignans (% SE)	0.59 ± 0.02 ^a^	0.83 ± 0.10 ^a^

^z^ Each value represents the mean ± standard error of three replicates. Mean values followed by different letters in their superscript are significantly different from each other (*p* = 0.05) in the respective row according to Duncan’s multiple range test.

**Table 5:** Fatty acid composition of *Ficus drupacea* pulp and fruit oil.

Fatty Acid	Chain Length		Pulp Oil	Seed Oil	
		RT (in min)	% Composition ^z^	RT (in min)	% Composition ^z^
Lauric acid	12:0	10.733	0.25 ± 0.02 ^a^	ND	ND
Myristic acid	14:0	12.251	0.61 ± 0.02 ^a^	12.251	0.12 ± 0.01 ^b^
Pentadecanoic acid	15:0	13.082	0.46 ± 0.01 ^a^	13.072	0.08 ± 0.01 ^b^
Palmitic acid	16:0	13.954	46.08 ± 0.29 ^a^	13.964	18.16 ± 0.12 ^b^
Palmitelaidic	16:1n9	14.384	0.52 ± 0.01 ^a^	14.384	0.14 ± 0.01 ^b^
Hexadecanoic acid, 14-methyl	14Me-16:0	14.877	0.59 ± 0.02 ^a^	ND	ND
Stearic acid	18:0	15.923	2.81 ± 0.06 ^b^	15.943	3.73 ± 0.09 ^a^
Oleic acid	18:1n9	16.395	7.07 ± 0.09 ^b^	16.426	12.63 ± 0.17 ^a^
Vaccenic acid	18:1n11	16.497	1.09 ± 0.02 ^a^	16.518	0.99 ± 0.06 ^a^
Linoleic acid	C18:2n9,12	17.256	28.61 ± 0.17 ^a^	17.297	26.81 ± 0.17 ^a^
Linolenic acid	18:3n9,12,15	18.426	11.91 ± 0.12 ^b^	18.487	36.87 ± 0.18 ^a^
Paullinic acid	20:1n13	ND	ND	19.195	0.11 ± 0.01 ^a^
Behenic acid	22:0	ND	ND	21.236	0.37 ± 0.01 ^a^
Total saturated fatty acids		NRV	50.80	-	22.46
Total monounsaturated fatty acids		NRV	8.68	-	13.87
Total polyunsaturated fatty acids		NRV	40.52	-	63.68

^z^ Each value represents the mean ± standard error of three replicates. ND = Not detected. NRV = No retention value. Mean values followed by different letters in their superscript are significantly different from each other (*p* = 0.05) in the respective row according to Duncan’s multiple range test.

**Table 6:** Phytochemical composition of *Ficus drupacea* fruit extracts.

Activity	Acetone (mg/g Extract) ^z^	Methanol (mg/g Extract) ^z^	Water (mg/g Extract) ^z^
Extract yield (mg/100 g DW)	20.86	259.98	3.96
Total phenolics (GAE)	60.12 ± 2.69 ^b^	25.78 ± 1.46 ^c^	70.50 ± 2.04 ^a^
Flavonoids (QE)	16.53 ± 1.03 ^a^	5.88 ± 0.53 ^b^	7.12 ± 0.37 ^b^
Alkaloids (AE)	3.76 ± 0.62 ^a^	1.37 ± 0.08 ^b^	0.04 ± 0.01 ^c^

^z^ Each value represents the mean ± standard error of three replicates. GAE—Gallic acid equivalent; QE—Quercetin equivalent; AE—Atropine equivalent. Mean values followed by different letters in their superscript are significantly different from each other (*p* = 0.05) in the respective row according to Duncan’s multiple range test.

**Table 7:** Antioxidant activities of *Ficus drupacea* fruit extracts.

Activity	Acetone (mg/g Extract) ^z^	Methanol (mg/g Extract) ^z^	Water (mg/g Extract) ^z^
DPPH (mg GAE)	3.81 ± 0.87 ^a^	2.54 ± 1.09 ^a^	4.82 ± 0.59 ^a^
TAA (mg AAE)	3103 ± 3.20 ^a^	97.99 ± 13.68 ^b^	139.15 ± 0.95 ^b^
FRAP (mg AAE)	965.21 ± 25.20 ^a^	10.66 ± 0.35 ^b^	38.14 ± 0.85 ^b^

^z^ Each value represents the mean ± standard error of three replicates. GAE—Gallic acid equivalent; AAE—Ascorbic acid equivalent. Mean values followed by different letters in their superscript are significantly different from each other (*p* = 0.05) in the respective row according to Duncan’s multiple range test.

## Data Availability

The original contributions presented in the study are included in the article, further inquiries can be directed to the corresponding author.
